# Comprehensive Analyses of the Bacterial Population in Non-Healing Claw Lesions of Dairy Cattle

**DOI:** 10.3390/ani12243584

**Published:** 2022-12-18

**Authors:** Kaoru Hori, Takako Taniguchi, Trigan Elpita, Rathanon Khemgaew, Satomi Sasaki, Yasuhiro Gotoh, Ichiro Yasutomi, Naoaki Misawa

**Affiliations:** 1 Graduate School of Medicine and Veterinary Medicine, University of Miyazaki, 5200 Kihara-kiyotake-cho, Miyazaki 889-1692, Japan; 2 Fuchu Veterinary Clinical Center, Hiroshima Agricultural Mutual Aid Association, 396-1 Fukae, Jyoge-cho, Fuchu, Hiroshima 729-3421, Japan; 3 Center for Animal Disease Control, University of Miyazaki, 1-1 Gakuenkibanadai-nishi, Miyazaki 889-2192, Japan; 4 Department of Bacteriology, Faculty of Medical Sciences, Kyushu University, 3-1-1 Maidashi, Higashi-ku, Fukuoka 812-8582, Japan; 5 Yubetsu Herd Management Service Ltd., 450-3 Baro, Mombetsu-gun, Yubetsu-cho 093-0731, Hokkaido, Japan

**Keywords:** bovine digital dermatitis, dairy cattle, *Fusobacterium necrophorum*, next-generation sequencing analysis, polymicrobial infection, *Treponema* species, non-healing

## Abstract

**Simple Summary:**

Non-healing claw lesions (NHCLs) are a newly characterized disorder affecting the deep dermis of the hoof in dairy cattle. Although NHCLs have been associated with bovine digital dermatitis (BDD), their precise etiology is not yet understood. To investigate the bacterial populations of NHCLs, 16S rRNA-based metagenomic analysis with next-generation sequencing (NGS) was employed. As reported in BDD, *Treponema* species and *Fusobacterium necrophorum* were detected frequently in NHCLs by PCR and immunohistochemistry, but NGS showed that both bacterial genera were not predominant in NHCLs. The predominant bacterial genera in NHCLs differed among the lesions examined, suggesting that the Treponema species present predominantly in BDD were not predominant in NHCLs and that the constituent bacterial population in NHCLs may vary among individual cattle and/or farms.

**Abstract:**

Non-healing claw lesions (NHCLs) are a newly characterized disorder affecting the deep dermis of the hoof in dairy cattle. Although NHCLs are thought to be associated with bovine digital dermatitis (BDD), their precise etiology is not yet understood. To investigate the bacterial populations present in each type of NHCL (toe necrosis: TN, non-healing white line disease: nhWLD, and a non-healing sole ulcer: nhSU), and the newly added entity non-healing verrucous-like lesions (nhVLL), 16S rRNA-based metagenomic analysis with next-generation sequencing (NGS) was employed. Twelve cases of NHCLs (3 TN, 3 nhWLD, 4 nhSU, and 2 nhVLL) were collected from five dairy farms in two prefectures in Japan. Three samples of healthy hoof dermis collected from two farms and a slaughterhouse were used as controls. Furthermore, culture-dependent and -independent approaches were conducted for detecting *Treponema* species and *Fusobacterium necrophorum*. As reported in BDD, *Treponema* species and *F. necrophorum* were detected frequently from NHCLs by PCR and immunohistochemistry, but NGS showed that these bacterial genera were not predominant in NHCLs. The predominant bacterial genera in NHCLs differed among the lesions examined, suggesting that *Treponema* species present predominantly in BDD were not predominant in NHCLs and that the bacterial population in NHCLs may vary among individual cattle and/or farms.

## 1. Introduction

In recent years, improvements in livestock and changes in feeding patterns in pursuit of higher performance and economic efficiency have led to breeding management that ignores the nutritional requirements of individual livestock animals. In addition, the supply of large amounts of concentrated feed has led to growth and production that exceeds the limits of livestock capacity. As a result, various diseases—referred to collectively as “production diseases”—have emerged [1]. Among the many known diseases, hoof disease of dairy cattle is one of the most significant [2].

In 2008, Cook and Birgy described a new hoof lesion that they termed “hairy attack” [3]. It is a disease that spreads along the white zone to the crown of the hoof on the dorsal wall and is considered a proliferative lesion of the white zone dermis. Holzhauer and Vos also reported “non-healing white line disorders” as a very intractable hoof disease [4]. Since then, clinicians and hoof trimmers have paid attention to these non-healing claw lesions (NHCLs). Since NHCLs cause severe pain, prolonged lameness, and are refractory to treatment, it is considered the most difficult hoof disease to cure and often leads to amputation of digits or slaughter [2,5]. Therefore, NHCLs cause significant economic losses due to a reduction in milk production and decreased reproductive performance and are also problematic from the viewpoint of animal welfare [2].

The pathogenesis of NHCLs is characterized by tissue necrosis from the hoof horn to the dermis associated with a distinctive foul odor and deep infection that can lead to osteolysis. NHCLs have been classified on the basis of the most frequent occurrence sites on the hoof. A typical example of NHCL is toe necrosis (TN), in which necrosis progresses from the hoof horn at the toe to the deeper dermis [6]. Another example is non-healing white line disease (nhWLD), in which necrosis progresses from the white line, which is vulnerable and easily accessible to infection, to a deeper level [3,4]. However, unlike white line disease, in which pus accumulates and tunnels are formed, the deep infection spreads to the surrounding area and extends to the bone. Furthermore, an NHCL occurs at the sole–hoof heel junction, a common site of sole ulcers, and the deep infection that causes severe necrosis is called a non-healing sole ulcer (nhSU) [6]. Furthermore, Blowy (2012) reported that ‘verrucous lesions’ and ‘honeycomb’ on the dorsal hoof wall, which extends into the dermis, create deep tissue infection and have a distinctive odor [5]. Since such lesions are characterized by recurrence after excision and are intractable, as described for other NHCLs, non-healing verrucous-like lesions (nhVLL) may possibly be categorized as NHCLs (Table 1).

The precise etiology of NHCLs has remained unclear. When Cook and Birgy reported “hairy attack”, they never referred to the etiology. However, this lesion resembles bovine digital dermatitis (BDD) that forms on the dermis of the white line. Blowely reported the occurrence of BDD in the exposed dermis of a non-healing hoof ulcer along with photographs [6] and described it as a secondary treponeme infection. Therefore, this might have represented an example of “hairy attack” or “non-healing” with spillover of BDD into the white line dermis. Since then, some researchers have reported that NHCLs may be associated with BDD because the dominant bacterial species present, such as *Treponema* spp., were detected by PCR. However, it is unclear whether these treponemes originate from BDD, since it was not described whether BDD was also present on the hoof where NHCL occurred [2,7,8]. On the other hand, Evans and Sykora have demonstrated that *Treponema* phylogroups may have a role in the pathogenesis of NHCLs [2,8], since NHCLs seemed to occur when there was a high rate of BDD on farms and that spirochetes were observed on direct smears [6,9]. The latter authors have also reported that *Treponema* phylogroups may play a pathogenic role in NHCLs [2,8] and that spiral-shaped rods have been observed in stamp preparations [6,10].

Culture-independent examinations of BDD lesions have shown that multiple *Treponema* species are consistently and predominantly found in both the superficial and deeper layers of the epidermis and that these treponemes are likely crucial agents in the pathogenesis of the disease [9,11,12]. However, various bacterial species have also been detected concomitantly [13,14,15,16]. Similarly, culture-based and culture-independent examinations have helped to reveal the bacterial population in NHCLs. As reported in BDD, *Treponema medium*, *Treponema phagedenis*, and *Treponema denticola* have been detected in NHCLs by PCR [2,8]. DNA amplicons of *Fusobacterium necrophorum* and *Porphyromonas endodontalis* have also been detected frequently, suggesting that NHCLs may be polymicrobial infections as has been reported for BDD. These findings are the basis for the claim that NHCLs are associated with BDD. However, even if treponemal infection is an accompanying feature, it remains unclear whether NHCLs are associated with BDD treponemal secondary infection since BDD does not usually occur inside the hoof, whereas NHCLs occur in the deep dermis.

To clarify more precisely the microbial populations present in NHCLs, unknown and yet uncultured organisms also need to be investigated, and quantitative analyses are also required to determine the bacterial composition of each NHCL. Since unculturable bacteria detected in BDD lesions may be involved in NHCLs [17], culture-based analyses of entire bacterial populations may be of limited value. Moreover, PCR detection of DNA from only a limited number of bacterial species is insufficient. To overcome this problem, a more comprehensive analysis of the bacterial populations in NHCLs would be desirable because 16S rRNA gene metagenomic sequencing has been shown to be useful for analyzing the bacterial populations of BDD lesions [18].

In the context of reports on the etiology of NHCLs, it is necessary to clarify whether treponemes and other bacteria detected in BDD lesions also play a role. To address this issue, quantitative and comprehensive analyses are needed to determine the bacterial composition in each type of NHCL. The objectives of the present study were (i) to isolate treponemes from NHCLs (nhWLD, nhSU, TN, and nhVLL), (ii) to detect DNA fragments and antigens of *T. phagedenis* and *F. necrophorum* in NHCLs using PCR and immunohistochemistry, respectively, and (iii) to perform 16S rRNA-based metagenomic analysis by next-generation sequencing (NGS) for detection of NHCL-associated microbiota.

## 2. Materials and Methods

### 2.1. Sample Collection

**Figure 1:**
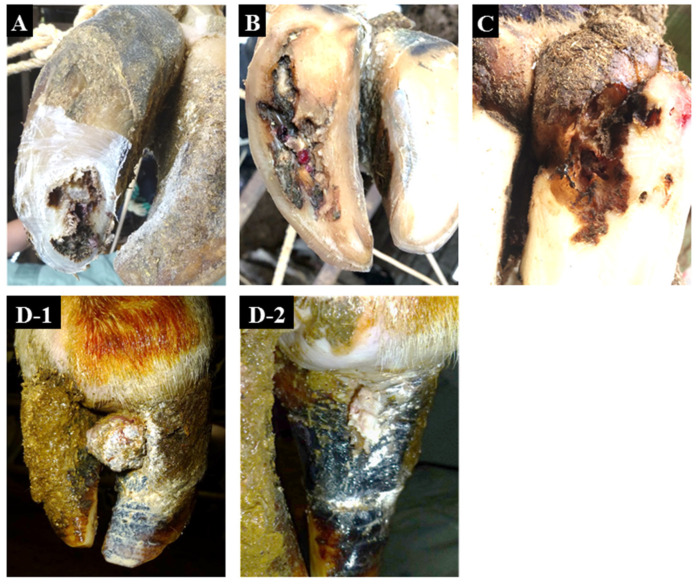
Representative non-healing claw lesions examined in this study. (**A**), toe necrosis (No. 3). (**B**), non-healing white line disease (No. 4). (**C**), non-healing sole ulcer (No. 7). (**D-1**), non-healing verrucous-like lesion (No. 12) before surgical excision. (**D-2**), non-healing verrucous-like lesion (No. 12) after surgical excision.

We sampled 12 NHCLs (3 TN, 3 nhWLD, 4 nhSU, and 2 nhVL) from 11 dairy cattle on 5 farms in Hiroshima Prefecture (A–C) and Hokkaido (F and G) of Japan from May 2016 to May 2019 (Table 2). Sample numbers 7 and 8 were collected from different hooves in the same cattle. Typical examples of the 4 categories of NHCLs examined are presented in Figure 1. Three samples of healthy foot skin from two dairy farms in Hiroshima Prefecture (D and E) were collected at a slaughterhouse after confirming that the cattle had been raised on NHCL-free farms. Farms A, B, F, and G are free-stall, while farms C, D, and E are tie-stall. Farms A, B, D, E, F, and G were fed a total mixed ration (TMR), and farm C was fed separate rations. Consistent with the incidence of hoof disease, the incidence of NHCL was also observed in a relatively small number of other cows on farms A, B, C, F, and G, but not on farms D and E, where control samples were taken. Regarding treatment history, the dairy cow in sample No. 3 was treated for NHCL by antibiotic application (oxytetracycline 500 mg) for the affected lesion 61 days before sampling. However, 61 days had elapsed since treatment, and furthermore, the clinical findings had not improved. It was therefore added to the sample as not being affected by the drug treatment. The other cows sampled had no history of NHCL treatment prior to sample collection. Regarding other hoof disease history, the dairy cow that sampled No. 5 was treated for WLD 83 days prior to sampling, but the other sampled cows had no other hoof disease history. Samples Nos. 7 and 8 were taken on the same day from different hind hooves of the same cattle with nhSU. Before sampling, the lesions were washed with tap water using a soft brush to remove fecal material. Biopsy samples were obtained using a 6 mm diameter sterile dermal biopsy punch or a sterile scalpel under sedation or local anesthesia. The biopsy samples were placed in Mitsuoka’s anaerobic buffer [19] in a sterilized tube, held at 4 °C, and transported to the laboratory at the University of Miyazaki within 48 h after sampling.

### 2.2. Isolation and Identification of Treponema *spp.*

PDDTp agar developed for the isolation of treponemes from BDD lesions [20] was used. PDDTp agar consists of GAM agar (Nissui Pharmaceutical Co., Tokyo, Japan) as a base medium for anaerobes supplemented with 0.8% brucella broth (Becton Dickinson and Company, Tokyo, Japan), 0.8% heart infusion broth (Nissui Pharmaceutical), 10% heat-inactivated fetal bovine serum (Invitrogen, CA, USA), 10% defibrinated horse blood (Nippon Biotest Laboratories, Tokyo, Japan), rifampin (1 μg/mL) (Nakarai Tesque, Kyoto, Japan), and enrofloxacin (1 μg/mL) (Sigma-Aldrich Japan, Tokyo, Japan). The NHCL sample was cut with a sterile scalpel and stamped onto PDDTp agar plates. Then, the plates were incubated at 37 °C for 14 days without oxygen in an AnaeroPack (Mitsubishi Gas Chemical Co., Tokyo, Japan). Film-like or swarming colonies were stained with Gram’s crystal violet solution (Sigma-Aldrich Japan) to observe the morphology. Identification of isolated spiral-shaped rod species was confirmed by the nucleotide sequences of the nearly complete 16S rRNA gene, as described elsewhere [20]. DNA was extracted by alkaline treatment (50 mM NaOH) followed by heat treatment at 95 °C for 5 min in accordance with the method reported by Misawa et al. [21]. The near-complete 16S rRNA gene (1500 bp) was amplified with the universal primers 8F (5′-AGAGTTTGATCMTGGCTCAG-3′) and 15R (5′-AAGGAGGTGATCCARCCGCA-3′) [22]. PCR was performed in a final volume of 20 µL containing the forward and reverse primers at 20 pM, each deoxynucleoside triphosphate at 200 µM, 0.5 U of Taq DNA polymerase (Qiagen, Tokyo, Japan), 1 × PCR buffer, and 20 ng of DNA template. Thermal cycling conditions were 95 °C for 2 min, followed by 25 cycles of denaturation at 95 °C for 30 s, annealing at 55 °C for 30 s, and extension at 72 °C for 2 min, with a final extension of 72 °C for 7 min. After amplification, the resulting amplicons were electrophoresed in 1% agarose gel. The PCR products were purified with a QIA quick PCR Purification Kit (Qiagen). The nucleotide sequence of each isolate was determined directly from the PCR fragment in a PCR-based reaction using ABI BigDye 3.1v (Applied Biosystems, Tokyo, Japan) and analyzed with a 3130 DNA sequencer (Applied Biosystems). A fragment of approximately 800 bp was sequenced from each 5′ and 3′ end of the amplicon, and the fragments were then assembled to determine the nucleotide sequence of the near-complete 16S rRNA gene (∼1500 bp) using serial cloner 2.6.1 software. Database similarity searches were carried out through the National Center for Biotechnology Information using the BLASTN algorithm. The partial nucleotide sequences of the 16S rRNA gene of *T. phagidenis* strains (Sample Nos. 1 and 7) were submitted to GenBank. The accession numbers of strains Nos. 1 and 7 were LC669686 and LC669687, respectively.

### 2.3. Phylogenetic Tree Analysis

ClustalW [23] was used to align the sequences of the 16S rRNA genes. The 16S rRNA gene sequences of *T. phagedenis* (accession nos. CP054692, FJ004921, KP063156, KP063178, KR025834, KR025835, KR025851, and MN396624), *T. denticola* (accession no. NC002967), *T. pedis* (accession no. EF061267), and *T. medium* (accession no. D85437) were obtained from the NCBI database. These sequence data were used for phylogenetic tree construction using the neighbor-joining method [24], and the topology of the tree was evaluated by 1000 bootstrap trials with the sequence boot program in MEGA-X.

### 2.4. Specific PCR Assays

The NHCL sample was cut into small pieces with a sterile scalpel and DNA was extracted using DNeasy Blood and Tissue Kits (Qiagen). PCR detection for *T. phagedenis* and *T. pedis* was conducted by the method of Beninger et al. [25] with some modifications. The primers employed were pha_F (5′-TCCGCCTACGACTGCGAT ACCA-3′) and pha_R (5′-CGGAACTGTCACAACTGGCGGA-3′) for *T. phagedenis*, ped_F (5′-TGGATGTTACGGAAGAGACACCGA-3′) and ped_R (5′-TGCCCCACTCTTACAAGTTCATCCCA-3′) for *T. pedis*. PCR was performed in a reaction mixture of 20 μL containing 1 μL of template DNA, 2 μL of 10× PCR buffer (Qiagen), 160 nM each primer, 0.625 U of Taq DNA polymerase (Qiagen), and 250 μM each deoxynucleotide triphosphate (Qiagen). The PCR for *T. phagedenis* and *T. pedis* comprised 35 cycles, with each cycle consisting of 94 °C for 30 s, 57 °C for 40 s, and 72 °C for 40 s. After the final cycle, the samples were heated at 72 °C for 5 min for the final extension reaction.

PCR detection of *T. denticola* and *T. medium* was conducted by the method of Asai et al. [26] with some modifications. The primers employed were den_F (5′-TAATACCGAATGTGCTCATTTACAT-3′) and den_R (5′-CTGCCATATCTCTATGTCATTGCTCTT-3′) for *T. denticola*, med_F (5′-CACTCAGTGCTTCATAAGGG-3′) and med_R (5′-CCGGCCTTATCTCTAAGACC-3′) for *T. medium*. The PCR reaction solution was prepared as described above. After an initial denaturation at 94 °C for 2 min, the PCR for *T. denticola* and *T. medium* was conducted for 30 cycles with each cycle consisting of 94 °C for 45 s, 62 °C for 1 min, and 72 °C for 1 min.

PCR detection of *F. necrophorum* was conducted by the method of Bennett et al. [27] with some modifications. The primers used in this assay, which target the lktA gene, were lktA-F (5′-ACAATCGGAGTAGTAGGTTC-3′) and lktA-R (5′-ATTTGGTAACTGCCACTGC-3′). The PCR reaction solution was prepared as described above and the PCR thermal profile consisted of denaturation at 94 °C for 2 min followed by 35 cycles of 94 °C for 30 s, 59 °C for 30 s, and 72 °C for 30 s, with a final extension step at 72 °C for 5 min.

After amplification, the resulting amplicons were electrophoresed in 1% agarose gel.

### 2.5. Immunohistochemical Staining

A total of 12 NHCL samples were examined by immunohistochemical staining for the presence of *T. phagedenis* and *F. necrophorum* antigens. Formalin-fixed, paraffin-embedded tissue sections (4–5 μm thick) were placed on glass slides coated with saline. Then, the sections were deparaffinized and autoclaved at 121 °C for 5 min. Endogenous peroxidase was inactivated for 10 min with methanol containing 3% H_2_O_2_. Next, the sections were treated with 10% BSA at 37 °C for 30 min and washed with PBS. Predetermined dilutions of each polyclonal rabbit antiserum, which were prepared as previously described [28], were applied to the sections and incubated at 37 °C for 1 h. Pooled sera collected from rabbits prior to immunization in this study were used as a negative control. After the slides had been washed, goat anti-rabbit immunoglobulins labeled with horseradish peroxidase (Envision polymer; Dako Japan, Kyoto, Japan) were applied to the sections, which were then incubated at 37 °C for 30 min and stained with 3,3’-diaminobenzidine (Dako Japan). The reaction was stopped by washing the slides in distilled water. The slides were then counterstained with hematoxylin and observed under a light microscope.

### 2.6. 16S rRNA Gene Amplicon-Based Metagenomic Analysis

10 NHCLs and 3 normal samples were tested for NGS analysis. DNA extracted from NHCL and normal samples was used as a template. The 16S rRNA amplicon libraries (V4 region) were prepared according to the Illumina 16S Metagenomic Sequencing Library Preparation protocol with the primers 515F (5′-TCGTCCGCCAGCGTCAGATGTGTATAAGAGACAG-GTGYCAGCMGCCGCGGTAA-3′) and 806R (5′-GTCTCGTGGGCTCGGAGATGTGTATAAGAGACAGGGACTACNVGGGTWTCTAAT-3′). The libraries were sequenced on the Illumina MiSeq platform to generate paired end (301 bp × 2) sequence reads. Phylogenetic analysis was performed using the Quantitative Insights into Microbial Ecology 2 (QIIME 2) platform version 2019.7 [29] Raw sequence data were demultiplexed denoised with DADA2 [30] using the parameters p-trim-left-f 10, p-trim-left-r 10, p-trunc-len-f 200, and p-trunc-len-r 150. Amplicon sequence variants (ASVs) were assigned taxonomy using the feature classifier classify-sklearn and the SILVA 132 99% 515F/806R reference sequences [31].

### 2.7. Data Deposition

The sequence data obtained in this study have been deposited at GenBank/EMBL/DDBJ under BioProject accession number PRJDB14531.

## 3. Results

### 3.1. Isolation of T. phagedenis from NHCLs

**Figure 2:**
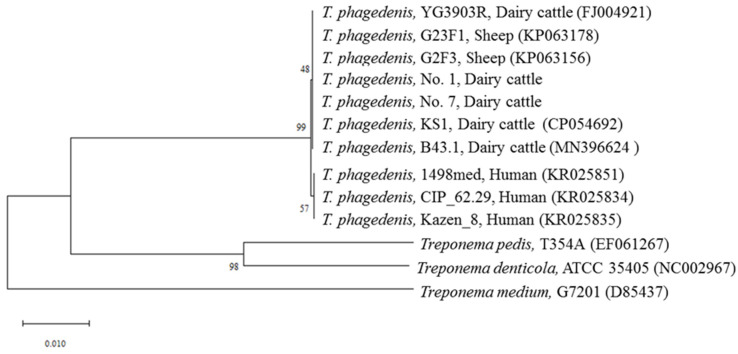
Phylogenetic tree analysis based on the nucleotide sequences of 16S rRNA genes.

Film-like or swarming colonies showing large spiral-shaped rods by crystal violet staining were detected in one TN (sample No. 1) and one nhSU (sample No. 7) among the 12 NHCL samples examined, and no treponeme was isolated from the normal dermis (Table 2). These isolates were identified as *T. phagedenis* based on the 16S rRNA gene sequences at high sequence similarities (100%) with those of *T. phagedenis* strain YG3903R which was isolated from a BDD lesion in Japan. Figure 2 shows a phylogenetic tree based on the nucleotide sequences of the 16S rRNA gene including these two isolates. The isolates belonged to the same cluster as the strains isolated from BDD of dairy cattle and sheep and to a cluster different from that of human strains.

### 3.2. PCR Detection

Table 2 shows the detection of PCR amplicons specific for *T. phagedenis*, *T. denticola*, *T. medium*, *T. pedis*, and *F. necrophorum*. PCR products of *Treponema* spp. were detected in 10 out of 12 NHCL samples (83.3%) and from all NHCL types even though the detection rate for each species differed among the samples examined. *F. necrophorum* DNA was detected in 11 NHCL samples (91.7%). Nucleotide sequencing of all amplicons of four *Treponema* species and *F. necrophorum* showed >99% similarity to each species deposited in the NCBI database (data not shown). In contrast, *Treponema* spp. and *F. necrophorum* were not detected from any samples of the normal dermis.

### 3.3. Immunohistochemical Analysis

**Figure 3:**
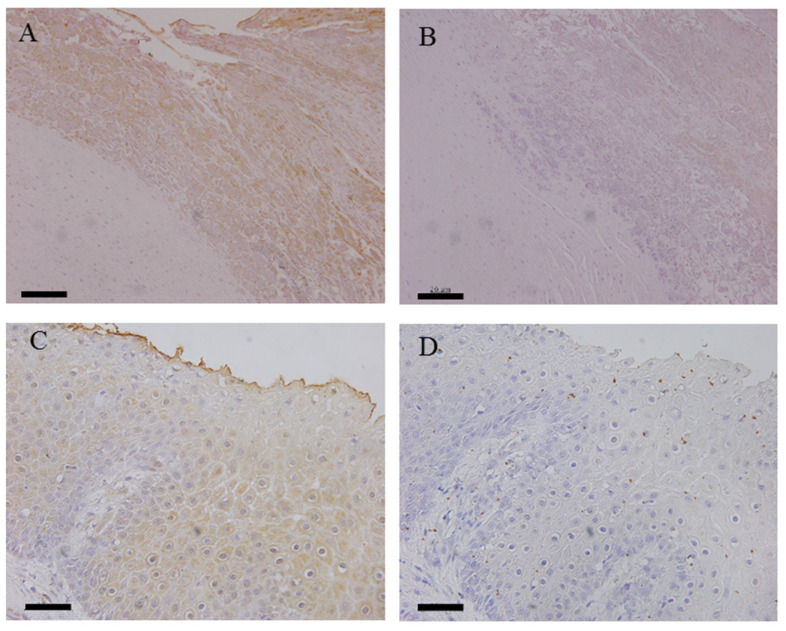
Detection of antigens of *T. phagedenis* and *F. necrophorum* by immunohistochemistry. (**A**), No. 2 (TN) using by rabbit polyclonal antibodies against T. phagedenis. (**B**), No. 2 (TN) using normal rabbit serum. (**C**), No. 9 (nhSU) using by rabbit polyclonal antibodies against *F. necrophorum*. (**D**), No. 9 (nhSU) using by normal rabbit serum. Bar = 20 μm.

Immunohistochemical examinations were carried out to determine whether the antigens of *T. phagedenis* and/or *F. necrophorum* were present in the samples of NHCLs and healthy foot dermis (Table 2). Antigens of *T. phagedenis* and *F. necrophorum* were detected in 8 out of 11 (72.7%) and 10 out of 12 (83.3%) NHCL tissues examined, respectively. Both antigens were detected in the surface layer of each lesion as weak signals (Figure 3). The antigens of *T. phagedenis* were not detected in any normal hoof tissues, but the antigens of *F. necrophorum* were detected in one healthy hoof dermis.

### 3.4. Bacterial Populations in NHCL by 16S rRNA Gene Amplicon-Based Metagenomic Analysis

To investigate the bacterial populations of the NHCLs, we analyzed the lesions and healthy hoof dermis by 16S rRNA-based amplicon analysis of the NHCL-associated microbiota. The preprocessing for filtering, denoising, merging, and non-chimeric step with QIIME2 yielded analyzable read counts of 386,714 for 13 samples (10 NHCL samples and 3 control samples) (14,202–37,069 reads per sample). The relative abundances at the genus levels of taxonomic classification were analyzed from the amplicon sequences.

**Figure 4:**
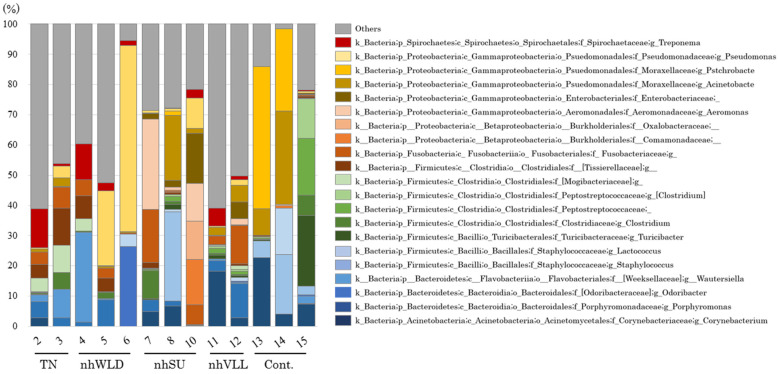
The 16S rRNA amplicon-based metagenomic analysis of non-healing lesion-associated microbiota.

There was no common predominant genus in the lesions overall or for each clinical condition, and all of the lesion samples showed a variety of bacterial population structures (Figure 4). In 10 lesions, the genus *Treponema* accounted for 0–12.8% of the population (4.0% on average). There was no *Treponema* in sample No. 7. The genus *Fusobacterium* was present in all lesions and accounted for 0.3–17.6% of the population (6.0% on average). In addition, the genus *Porphyromonas* was also present in all lesions, and accounted for 0.035–11.3% of the population (3.9% on average). Only the genera *Fusobacterium* and *Porphyromonas* were commonly present in all lesions. All three genera were also present in control No. 15: *Treponema* 0.4%, *Fusobacterium* 0.3%, and *Porphyromonas* 0.1%.

Most of the lesion samples had a higher abundance of the minor population (<8%) than the control samples, especially TN and nhVLL. *Pseudomonas* was commonly predominant in samples Nos. 5 and 6, which had been collected from farms F and G in Hokkaido, but *Pseudomonas* was not present in sample No. 4, which was also categorized as nhWLD, from farm C in Hiroshima Prefecture. Although samples Nos. 7 and 8, categorized as nhSU, had been collected on the same day from different hind hooves of the same dairy cattle, their bacterial compositions differed, as was also the case of nhSU lesions of different cattle (sample No. 10) (Figure 4).

## 4. Discussion

The concept of infectious diseases caused by specific microbial populations, going one step beyond that of diseases caused by simultaneous multiple pathogens (i.e., complicated infectious diseases), has gradually been recognized. The term polymicrobial infection (PI) has been used for such diseases [32]. In the medical field, periodontal disease is known as a representative PI [33], but the pathogenic microorganisms involved are limited to those that can be cultured, thus limiting a full understanding of the extremely complex pathogenesis of the disease. Typical PIs that have become problematic in the veterinary field include bovine and porcine respiratory disease syndrome [32]. Based on culture-dependent and -independent analyses, BDD and NHCLs may also be categorized as PIs. However, Koch’s postulates for identifying the causative microorganisms have not been established for these infectious diseases, and even if the pathogens involved can be identified, a comprehensive analysis of the factors leading to the establishment of infectious diseases has yet to be completed.

The recent rapid progress in microbial genome analysis, has provided a wealth of whole-genome information on major pathogenic microorganisms, and research on the individual pathogens has intensified. Furthermore, the advent of NGS has yielded genomic information on microbial populations through metagenome analysis. At the same time, there has been renewed interest in the importance of studying the nonculturable microorganisms that exist in microbial populations. Therefore, in the present study, we conducted a comprehensive NGS-based analysis to clarify the etiology of NHCLs, as well as conducting conventional PCR and culture-based examinations.

It is considered that the pathogenesis of white line disease (WLD) and sole ulcers (SU) is a spillover from laminitis or physical damage [34]. On the other hand, it is still controversial whether the “non-healing” NHCL is a secondary severe deep infection of TN, SU, or WLD, or whether it is a completely different disease. Based on our experience with NHCL in clinical practice, we began this study to investigate the possibility of an independent pathology because NHCL can occur in any limb and internal or external hoof, and most affected cows with non-healing NHCL with infection have no history of TN, SU, or WLD. In our experience, only one nhWLD case had a previous history of WLD, which was recognized 81 days earlier. If the bacteria had infected the WLD or SU and transitioned to non-healing, then these cows would have had a history of WLD or SU. However, no such case was observed in the cattle examined in this study. Furthermore, there was no evidence of laminitis in non-healing, and non-healing occurs regardless of limb or internal or external hoof site, which is another major difference from the occurrence of WLD and SU. Therefore, in this manuscript, we would like to mention the possibility of infectious pathologies other than TN, SU, and WLD.

Culture-based examination of NHCLs isolated *T. phagedenis* strains from two specimens (TN and nhSU). Although the isolation rate was low (14.3%), this was the first recorded case of *T. phagedenis* culture from NHCLs (Table 2). Nucleotide sequencing of the 16S rRNA gene showed that all of the isolated strains had 100% identity with those of the *T. phagedenis* strain YG3903R isolated from a BDD lesion in Japan and other *T. phagedenis* strains [17]. Phylogenetic analysis based on the nucleotide sequences of 16S rRNA genes showed that both of the *T. phagedenis* strains isolated from NHCLs belonged to the same cluster, including strains isolated from BDD lesions (Figure 2), implying that *T. phagedenis* strains isolated from NHCLs are associated with those isolates from BDD lesions. Furthermore, PCR amplicons using primer sets for *T. phagedenis*, *T. denticola*, *T. pedis*, and *T. medium* were detected in 10 out of 12 samples (83.3%) with different detection rates, regardless of NHCL types (Table 2). The nucleotide sequences of the PCR amplicons of the four *Treponema* spp. showed high similarity (>99%) with the identical *Treponema* spp., suggesting the presence of multiple *Treponema* spp. in the lesions. Similarly, a DNA fragment of *F. necrophorum* was detected in 11 of the 12 samples (91.7%) examined by PCR. These results agreed with previous reports [2,7,8].

The results of this study, together with previous reports, appear to suggest that NHCLs may be associated with BDD. However, previous reports did not provide quantitative data on the bacterial species present in the lesions even though they showed high detection rates. PCR detection may misinterpret the nature of the key constituent species, resulting in non-reproducible findings in studies of etiology. For these reasons, we carried out detection of *T. phagedenis* and *F. necrophorum* antigens in NHCLs using immunohistochemistry and comprehensive analysis of the bacterial populations in different types of NHCL affecting dairy cattle.

Although antigens of *T. phagedenis* and *F. necrophorum* were detected in 8 out of 11 (72.7%) and 10 out of 12 (83.3%) NHCLs, respectively, the signals of both bacterial antigens were weak and detected only in the superficial layer of the hoof dermis. This suggested that *T. phagedenis* and *F. necrophorum* might be present in the lesions but that the number of bacteria might be low and not invade into the deeper dermis.

To investigate the differences in the bacterial populations of each type of NHCL and normal dermis, 16S rRNA-based metagenomic analysis with NGS was employed. In this dataset, we identified a total of 386,714 counts from 13 samples (14,202–37,069 reads per sample). Although the analyses showed that the genera *Treponema*, *Fusobacterium*, and *Porphyromonas* were detected frequently in the NHCL samples examined, their numbers were low relative to other bacterial genera, resulting in a variety of bacterial population structures and no predominant bacteria in any types of NHCLs (Figure 4).

Using 16S rRNA-based amplicon metagenomic analysis of BDD lesions, Gotoh et al. showed that *Treponema* was the most common pathogen, with operational taxonomic units (OTUs) accounting for 13.0–92.6% (mean 52.2%) of the total [18]. In contrast, in the present study, the genus *Treponema* accounted for 0–12.8% of the population (4.0% on average) in 10 lesions, and no *Treponema* was detected in sample No. 7. These results suggested that *Treponema* in NHCLs may not be the most predominant species in the affected hoof dermis. Although *T. phagedenis* was isolated from sample No.7, its DNA was not detected by PCR (Table 2). This may be due to the different lesion sites examined, but this fact also suggests that *Treponema* spp. may not be throughout the lesion. Furthermore, NHCLs have a large number of minor bacterial groups with a composition ratio of less than 8%, suggesting a very complex population comprising a wide variety of bacteria.

The present results suggested that bacteria present predominantly in BDD were not predominant in NHCLs and that the constituent bacterial population in NHCLs may depend on individual cattle and/or farm environment. Interestingly, samples Nos. 7 and 8 with nhSUs were collected from different hooves of the same individual on the same day, but the bacterial constituents of each lesion were quite different. A possible explanation for this difference may be that the same NHCL on different hooves may be at different stages of disease. It is also possible that the condition of the hoof may differ among the extremities of the same individual.

Furthermore, the bacterial populations of nhWLD in samples Nos. 4 (collected in Hiroshima Prefecture), 5, and 6 (collected in Hokkaido Prefecture) were also different despite the fact that the type of NHCL was the same. These findings are informative regarding the possible mechanism of pathogenesis, i.e., the fact that *Treponema* spp., *F. necrophorum*, and *Prorphylomonas* spp. were detected at high rates but were not the predominant bacteria suggests that these bacteria may act as a foothold in the early stages of infection and that the subsequent bacterial species developing within the lesions may depend on the rearing environment at each farm and the individual circumstances of the cattle.

Using NGS analysis of surface swab samples of claw horn disruption lesions (CHDLs), Bay et al. found that *Porphyromonas* spp., *Fusobacterium* spp., *Treponema* spp., Clostridiales Family XI, *Fastidiosipila* spp., *Peptoniphilus* spp, Ruminococcaceae UCG-014, uncultured Bacteroides, and other genera of the family Porphyromonadaceae were overrepresented even though the presence of *Porphyromonas* spp. and *Fusobacterium* spp. was consistent with the results. They suggested that many of the bacteria present might be secondary contaminants or opportunistic agents that had originally been present on the surface of the lesions and collected by swabbing [35]. Since we collected samples from the dermis inside the lesions, this difference in the testing methods might have affected the results.

Although high proportions of *F. necrophorum*, *Porphylomonas* spp., and *Pseudomonas* spp. were detected from NHCLs in the present study, they were not always the predominant species in all lesions. Since these bacteria are well-known opportunistic pathogens [36,37], it is unclear whether *Treponema* causes the primary infection and the other bacteria the secondary infection. However, it is likely that complex factors are involved in the pathogenesis of NHCLs. Furthermore, since NHCLs appear to occur repeatedly on the same farm in some cases and not exclusively on any particular farm, the pathogenesis of NHCLs would involve host, environmental, and husbandry conditions in addition to pathogenic bacteria.

Since NHCLs are not simple infections that satisfy Koch’s postulates, it is not easy to identify the true causative agent(s). Therefore, NHCLs may show a range of the pathogenicity, and the etiology may vary. For a better understanding of the pathogenicity and etiology of NHCLs, quantitative detection of the predominant bacteria within lesions by longitudinal analysis will be necessary. In addition, it will be important to investigate whether recent farming conditions, such as feeding management and hoof trimming, also play a role in the pathogenesis of this complex and intractable hoof disease in dairy cattle.

## 5. Conclusions

*Treponema* species and *Fusobacterium necrophorum* were detected frequently in NHCLs by PCR and immunohistochemistry, but NGS analysis showed that these bacterial genera were not predominant and that a large number of minor bacterial groups were also present. The constituent bacterial population in NHCLs may depend on the individual cattle and/or farms examined. The present findings suggest that NHCLs can be categorized as polymicrobial infections. In order to clarify the pathogenesis of NHCLs, it will be essential to identify the multiple pathogens involved and to clarify the factors that establish a comprehensive infection, including the host response and farm environment.

## Figures and Tables

**Table 1:** A typical example of NHCLs.

Condition	Affected Limb/Claw	Zones Affected	Characteristic Signs	Common Signs
toe necrosis (TN)	all limb, lateral/medial claw	sole at the toe	necrosis progresses from the hoof horn at the toe to the deeper dermis and bone	distinctive odor
non-healing white line disease (nhWLD)	all limb, lateral/medial claw	white line	necrosis progresses from the white line to a deeper level which spreads to the surrounding area and extends to the bone	distinctive odor
non-healing sole ulcer (nhSU)	all limb, lateral/medial claw	sole–heel junction	necrosis progresses from the sole–heel junction to a deeper level which spreads to the surrounding area and extends to the bone	distinctive odor
non-healing verrucous-like lesions (nhVLL)	all limb, lateral/medial claw	dorsal hoof wall	verrucous-like lesions occur in the dermis within the dorsal hoof wall and appear by lacerating the dorsal hoof wall	distinctive odor

**Table 2:** Culture isolation, PCR detection, and immunohistochemistry of *Treponema* spp. and *F. necrophorum*.

Sample No.	Farm	Lesion Type	Isolation	PCR					Antigen	
			*T*. *phagedenis*	*T*. *phagedenis*	*T*. *denticola*	*T*. *medium*	*T*. *pedis*	*F*. *necrophorum*	*T*. *phagedenis*	*F*. *necrophorum*
1	A	TN	+	+	-	+	+	+	+	-
2	B	TN	-	+	-	-	+	+	+	+
3	C	TN	-	+	+	+	+	+	+	+
4	C	nhWLD	-	+	+	+	+	+	+	+
5	F	nhWLD	-	+	+	+	+	+	+	+
6	G	nhWLD	-	+	-	-	+	+	-	+
7 *	C	nhSU	+	-	-	-	+	+	+	+
8 *	C	nhSU	-	-	-	-	-	-	-	+
9	C	nhSU	-	+	-	-	+	+	ND	+
10	G	nhSU	-	-	-	-	-	+	-	-
11	B	nhVLL	ND	+	+	+	+	+	+	+
12	B	nhVLL	-	+	+	+	+	+	+	+
13	D	Control	-	-	-	-	-	-	-	-
14	D	Control	-	-	-	-	-	-	-	-
15	E	Control	-	-	-	-	-	-	-	+

TN: toe necrosis; nhWLD: nonhealing white line disease; nhSU: non-healing sole ulcer;.nhVLL: non-healing verrucous-like lesion; ND: not done; * Different hooves in the same dairy cattle.

## Data Availability

Not applicable.
